# Almond population genomics and non-additive GWAS reveal new insights into almond dissemination history and candidate genes for nut traits and blooming time

**DOI:** 10.1093/hr/uhad193

**Published:** 2023-09-25

**Authors:** Felipe Pérez de los Cobos, Eva Coindre, Naima Dlalah, Bénédicte Quilot-Turion, Ignasi Batlle, Pere Arús, Iban Eduardo, Henri Duval

**Affiliations:** Fruticultura, Institut de Recerca i Tecnologia Agroalimentàries (IRTA), Mas Bové, Ctra. Reus-El Morell Km 3,8 43120 Constantí Tarragona, Spain; Centre de Recerca en Agrigenòmica (CRAG), CSIC-IRTA-UAB-UB. Institut de Recerca i Tecnologia Agroalimentàries (IRTA), Cerdanyola del Vallès (Bellaterra), 08193 Barcelona, Spain; Centre for Research in Agricultural Genomics (CRAG) CSIC-IRTA-UAB-UB, Campus UAB, Edifici CRAG, Cerdanyola del Vallès (Bellaterra), 08193 Barcelona, Spain; INRAE, GAFL, F-84143, Montfavet, France; INRAE, GAFL, F-84143, Montfavet, France; INRAE, GAFL, F-84143, Montfavet, France; Fruticultura, Institut de Recerca i Tecnologia Agroalimentàries (IRTA), Mas Bové, Ctra. Reus-El Morell Km 3,8 43120 Constantí Tarragona, Spain; Centre de Recerca en Agrigenòmica (CRAG), CSIC-IRTA-UAB-UB. Institut de Recerca i Tecnologia Agroalimentàries (IRTA), Cerdanyola del Vallès (Bellaterra), 08193 Barcelona, Spain; Centre for Research in Agricultural Genomics (CRAG) CSIC-IRTA-UAB-UB, Campus UAB, Edifici CRAG, Cerdanyola del Vallès (Bellaterra), 08193 Barcelona, Spain; Centre de Recerca en Agrigenòmica (CRAG), CSIC-IRTA-UAB-UB. Institut de Recerca i Tecnologia Agroalimentàries (IRTA), Cerdanyola del Vallès (Bellaterra), 08193 Barcelona, Spain; Centre for Research in Agricultural Genomics (CRAG) CSIC-IRTA-UAB-UB, Campus UAB, Edifici CRAG, Cerdanyola del Vallès (Bellaterra), 08193 Barcelona, Spain; INRAE, GAFL, F-84143, Montfavet, France

## Abstract

Domestication drastically changed crop genomes, fixing alleles of interest and creating different genetic populations. Genome-wide association studies (GWASs) are a powerful tool to detect these alleles of interest (and so QTLs). In this study, we explored the genetic structure as well as additive and non-additive genotype–phenotype associations in a collection of 243 almond accessions. Our genetic structure analysis strongly supported the subdivision of the accessions into five ancestral groups, all formed by accessions with a common origin. One of these groups was formed exclusively by Spanish accessions, while the rest were mainly formed by accessions from China, Italy, France, and the USA. These results agree with archaeological and historical evidence that separate modern almond dissemination into four phases: Asiatic, Mediterranean, Californian, and southern hemisphere. In total, we found 13 independent QTLs for nut weight, crack-out percentage, double kernels percentage, and blooming time. Of the 13 QTLs found, only one had an additive effect. Through candidate gene analysis, we proposed *Prudul26A013473* as a candidate gene responsible for the main QTL found in crack-out percentage, *Prudul26A012082* and *Prudul26A017782* as candidate genes for the QTLs found in double kernels percentage, and *Prudul26A000954* as a candidate gene for the QTL found in blooming time. Our study enhances our knowledge of almond dissemination history and will have a great impact on almond breeding.

## Introduction

One of the landmarks of human history was the transition from nomadic hunter–gatherer societies to settled agriculture-based societies. This transition, known as the Neolithic Revolution, marked the beginning of the domestication of wild plant species as cultivated crops [1]. Domestication, and later dispersal and diversification of crops, introduced substantial changes into their genomes, fixing alleles of interest, creating different genetic populations, and adapting these groups to different environmental conditions [2, 3]. These changes, accumulated over thousands of years, led to the crops we consume today.

Nowadays, the main actor changing crop genomes is modern breeding. The efficient implementation of breeding strategies requires the correct management of germplasms, optimized genotyping and phenotyping methods, concise knowledge of crop genetic structure, and the study of genetic determinism behind traits of interest [4, 5]. Genome-wide association study (GWAS) is a powerful tool to study quantitative traits in plant breeding. It aims to find polymorphic genetic markers (typically SNPs) significantly associated with phenotypic variation [6]. However, one of the weaknesses of this technique is that most GWAS models assume that the genotypic variation has an additive effect on the phenotype. This means that non-additive effects, such as dominant-recessive or overdominant interactions, are not included in the models even when they may be relevant for most traits [7].

Almond [*Prunus dulcis* Miller (D.A. Webb)] is the most economically important temperate nut tree worldwide. In the period 2011 to 2021, its production increased 54%, reaching 1 684 395 metric tons of kernel [8]. It belongs to the *Rosaceae* family and the *Prunus* genus with other important crops, including peach, plum, apricot, and cherry.

While research on the almond domestication process is in the early stages, some important insights have been made. The most accepted theory to date is that almond originated from hybridizations with several wild relatives somewhere between the Eastern Mediterranean and Southwest Asia, expanding rapidly to Central Asia and the Western Mediterranean. Many studies using different approaches support this theory, from analyses based on morphology, habitat, and/or coexistence in cultivated areas [9–11], through genomic analyses [12–14], to archaeobotanic evidence [15–17]. In this sense, many efforts have focused on analyzing the population structure of different almond germplasms [18–20]. Nevertheless, these studies have been limited by the geographical origin of the accessions (most accessions came from the same region) or by the low number of markers used. As a result of these drawbacks, our knowledge of the genetic structure of the cultivated almond is still limited.

**Figure 1:**
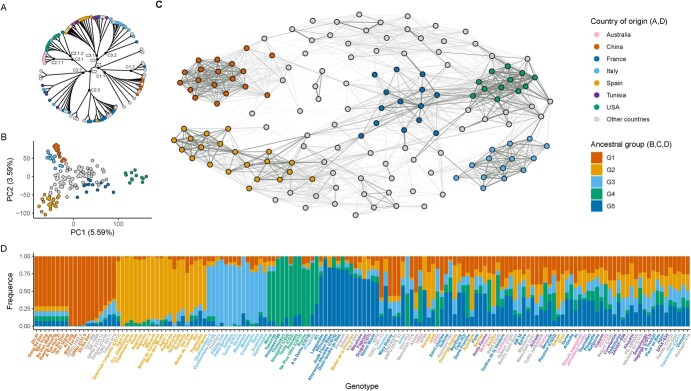
Genetic structure analysis. A) Phylogenetic tree. B) Principal components analysis C) Additive kinship. Edges with absolute weight less than 0.05 are not represented. D) Population structure analysis.

The recent publication of three almond reference genomes [21–23] and the development of a 60 K SNP array [24] have opened the door to performing genome-wide analyses on almond. So far, there have been three GWASs using genome-wide marker data [19, 20, 25], and several QTLs linked to shell and kernel quality traits were identified. Nevertheless, these studies only focused on additive GWAS models and the variability of the plant material was reduced. Studying a broader germplasm and analyzing non-additive genotype–phenotype associations would allow the exploration of the origin and historic dissemination of the cultivated almond at the same time that would help to find alleles of interest and QTLs fixed over thousands of years of domestication.

In this study, we explored the genetic structure and genotype–phenotype associations in a collection of 243 almond accessions from different origins. For this purpose, we first characterized the genetic diversity of the collection using the almond 60 K SNP array. Then we carried out a GWAS using additive and non-additive models for different traits, including kernel and nut weight, crack-out percentage, double kernels percentage, and blooming time. As far as we know, this is the first non-additive GWAS in Rosaceae species. Using candidate gene analysis, we also proposed candidate genes responsible for the main QTLs found in this analysis.

## Results

### Almond genetic structure defined five ancestral groups

The results from the population structure analysis, additive kinship, phylogenetic tree, and PCA indicated that the most acceptable genetic structure of the 152 landraces included in this analysis is a model with five ancestral populations (Figure 1). According to the population structure analysis (Figure 1D), 80 accessions were part of one of the five ancestral groups, while 72 were considered admixed. The number of accessions was homogeneous between groups, ranging from 12 to 21 accessions (G4 and G2 respectively). Ancestral group G1 was mainly composed by Asian accessions: 12 Chinese, two Iranian, two Turkish, and one Pakistani. This group also included one Greek and one Romanian accession. Ancestral group G2 was entirely formed by Spanish accessions. Ancestral group G3 was formed by 13 Italian accessions and one Greek. In ancestral group G4, nine of 12 accessions were from the USA, along with two French and one Italian accession. Ancestral group G5 had six French accessions, three Tunisian, one Greek, one Jordanian, one Iranian, one Moroccan, and one Spanish accession.

Additive kinship results showed five dense clusters (Figure 1C). These clusters included all the accessions forming the five ancestral groups from the population structure analysis. Clusters formed by G1 and G3 had less connections with other accessions. On the other hand, clusters formed by G2, G4, and G5 accessions had several connections between them and accessions from other countries. G2 accessions, separated in two sub-clusters, were also connected with accessions from Australia and North Africa, among others. G4 and G5 clusters were strongly connected, and the G5 cluster was connected to several accessions from Australia.

The phylogenetic tree had three main clades: C1, C2, and C3 (Figure 1A). C1, mainly formed by Asian accessions, was subdivided in two secondary clades, C1.1 and C1.2. All the Chinese accessions were situated in C1.1. C2 was mainly formed by French, American, and Australian accessions. American and Australian accessions were found in the same secondary clade, C2.1, but separated in two tertiary clades, C2.1.1 and C2.1.2. French accessions were in two different secondary clades, C2.2 and C2.3, along with some Tunisian, Italian and Spanish accessions. The Spanish, Italian and Tunisian accessions were in C3. And while the Spanish and Tunisian accessions were found in the same secondary clade, C3.1, most of the Italian accessions formed another secondary clade, C3.2.

Based on the Principal Compound Analysis, the ancestral groups were well separated according to the first two principal components, explaining 9.18% of the overall variation (Figure 1B). Admixture accessions were mostly in the center of the scatter plot with values close to 0 for the two principal components. Genotypes from G4 group were located to the right of the X axis while G1, G2, and G3 groups were to the left of the X axis and separated along the Y axis.

### Only three breeding cultivars had a F2 value over 0.4

**Figure 2:**
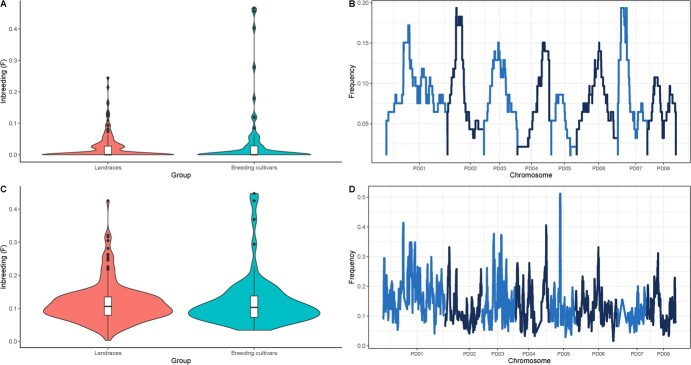
Homozygosity analysis. A) Inbreeding using F2. B) Freq2 results. C) Inbreeding using F0.25. D) Freq0.25 results.

Landraces and breeding cultivars had low levels of inbreeding using *F_2_* or *F_0.25_* values (Figure 2A and 2C). In the case of *F_2_*, used to detect recent inbreeding events, inbreeding was low for both landraces and breeding cultivars. Most of the cultivars had an *F_2_* value equal to zero, with only nine landraces and seven breeding cultivars exceeding *F_2_* = 0.1.

In the case of breeding cultivars, there were some exceptions with high levels of inbreeding, such as ‘Ayles’, ‘Amandier rose’, and ‘Garfi’, with *F_2_* values over 0.4. Using *F_0.25_* as the inbreeding measure, the inbreeding values of both landraces and breeding cultivars were slightly higher, with a mean *F_0.25_* around 0.1 in both cases. Only accessions ‘Ayles’, ‘Amandier rose’, and ‘DPRU 487-A’ had a *F_0.25_* value over 0.4.

Comparing *Freq_2_* and *Freq_0.25_* there were large differences (Figure 2B and 2D). No SNP had a *Freq_2_* higher than 0.2, while there were three genomic regions with *Freq_0.25_* higher than 0.4, in chromosomes 1, 4, and 5. In the case of PD01 and PD05, the genomic regions with high *Freq_0.25_* contained 24 and 27 genes, respectively, according to the ‘Texas’ reference genome v2.0 [22]. In contrast, the genomic region with high *Freq_0.25_* in PD04 contained only one gene, *Prudul26A014015*.

**Figure 3:**
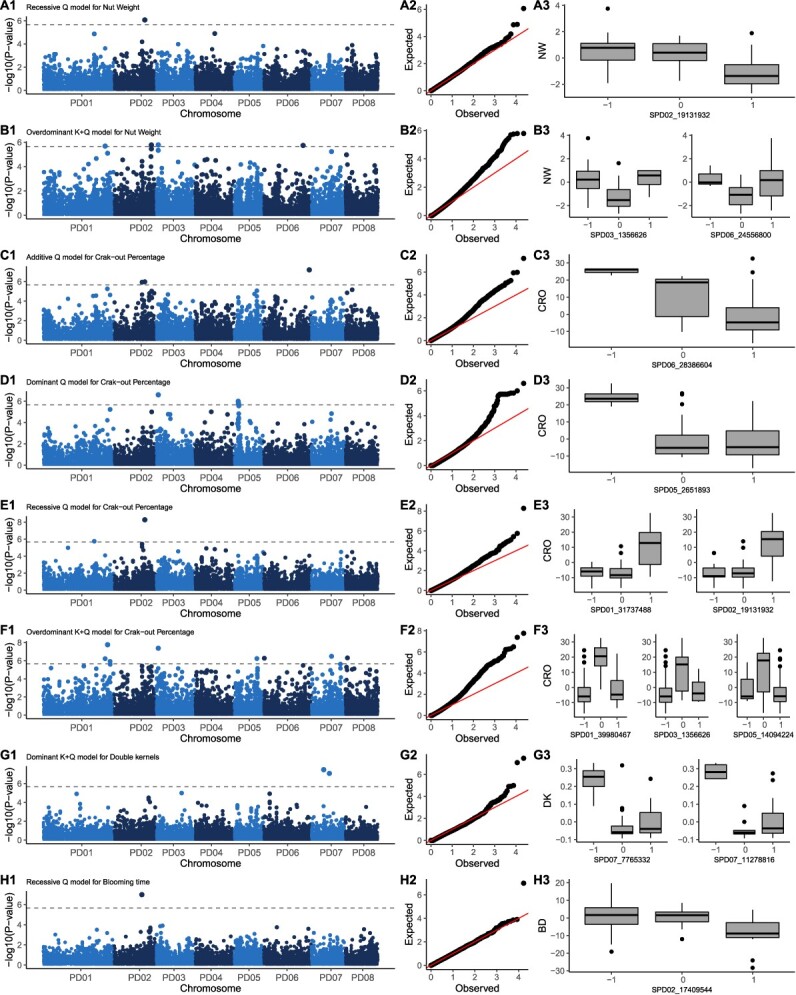
Genome-wide association analysis. Row A) Recessive Q model for Nut weight. Row B) Over-dominant K + Q model for Nut weight. Row C) Additive Q model for Crack-out percentage. Row D) Dominant Q model for Crack-out percentage. Row E) Recessive Q model for Crack-out percentage. Row F) Over-dominant K + Q model for Crack-out percentage. Row G) Dominant K + Q model for Double kernels percentage. Row H) Recessive Q model for blooming time. Column 1) Manhattan plots. Column 2) Q-Q plots. Column 3) Boxplots of the true positive QTLs.

### Linkage disequilibrium decay was between 4259 and 6904 bp

The mean size of the LD block was 6061 bp and ranged from 4259 bp (PD08) to 6904 bp (PD02) (Supplementary Material 1).

### All the traits had heritability higher than 0.90

A significant negative correlation was found between NW and CRO (−0.77) (Supplementary Material 1). A weaker correlation was also found between NW and KW (0.49). Genetic variance and the variance due to the environment (residual error) were extracted from the linear mixed models for each trait (Table 1). Heritability for all traits was higher than 0.90.

**Table 1:** Partition of variance of the traits under study

	KW	NW	CRO	DK	BLO
Genotype	0.024	2.05	136.62	0.0098	59.91
Error	0.023	0.43	9.27	0.0038	19.59
Number of repetitions	12.24	12.33	12.24	9.45	4.05
Heritability	0.93	0.98	0.99	0.96	0.93

NW: nut weight, CRO: crack-out percentage, DK: double kernels percentage, BLO: blooming time, VE: Variance explained, CVE: Combined variance explained.

### 13 true positive QTLs were found for the traits under study

In total, 19 associations were detected for the traits under study (Figure 3). Of these associations, six were considered false positives, since the phenotypic data distribution did not match the genotype–phenotype interaction searched. Therefore, 13 QTLs were considered true positives (Figure 3, column 3). These QTLs were named according to the recommendations for standard QTL nomenclature and reporting of the Genome Database for Rosaceae (Table 2).

**Table 2:** Summary of the QTLs identified, indicating the trait and the QTL, the genetic effect, the correction model used, the closest SNP and its chromosome location, the p-value, the variance explained and the combined variance explained

**Trait**	**Name**	**Effect**	**Correction**	**Top SNP**	**Chr**	**-log10(p-value)**	**VE**	**CVE**
**NW**	**qP-NW2**	Recessive	Q	SPD02_19131932	2	6.07	33.71%	45.74%
	**qP-NW3**	Overdominant	K + Q	SPD03_1356626	3	6.13	18.77%	
	**qP-NW6**	Overdominant	K + Q	SPD06_24556800	6	6.1	9.04%	
**CRO**	**qP-CRO6**	Additive	Q	SPD06_28386604	6	7.19	22.66%	71.78%
	**qP-CRO5.1**	Dominant	Q	SPD05_2529870	5	6	23.22%	
	**qP-CRO1.1**	Recessive	Q	SPD01_31737488	1	5.75	47.5%	
	**qP-CRO2**	Recessive	Q	SPD02_19131932	2	8.28	49.13%	
	**qP-CRO1.2**	Overdominant	K + Q	SPD01_39980467	1	7.76	33.44%	
	**qP-CRO3**	Overdominant	K + Q	SPD03_1356626	3	7.37	27.9%	
	**qP-CRO5.2**	Overdominant	K + Q	SPD05_14094224	5	6.23	23.57%	
**DK**	**qP-DK7.1**	Dominant	K + Q	SPD07_7765332	7	7.46	30.7%	65.87%
	**qP-DK7.2**	Dominant	K + Q	SPD07_11278816	7	7.54	47.57%	
**BLO**	**qP-BLO2**	Recessive	Q	SPD02_17409544	2	6.99	16.44%	-

K: kinship; Q: population structure, NW: nut weight, CRO: crack-out percentage, DK: double kernels percentage, BLO: blooming time, VE: Variance explained, CVE: Combined variance explained.

Within these 13 QTLs, only qP-CRO6 had an additive effect, the rest had non-additive effects on the phenotype. By trait, we found three, seven, two and one QTL for NW, CRO, DK and BLO, respectively. No QTL was found for KW. NW and CRO had QTLs located at the same position: qP-NW2 and qP-CRO2 had SPD02_19131932 as top SNP with a recessive effect, while qP-NW3 and qP-CRO3 had SPD03_1356626 as top SNP with a overdominant effect. The variance explained by the QTLs ranged from 9% (qP-NW6) to 49% (qP-CRO2). The combined variance explained for all the QTLs was 45.74% for NW, 71.78% for CRO, 65.87% for DK, and the only QTL found for BLO explained 16.44% of the variance.

### Candidate genes controlling crack-out percentage, double kernels percentage, and blooming time

The length of the qP-CRO2 region was 13.8 kb, containing just one gene (Table 3). This gene, *Prudul26A013473*, was annotated as a NAC domain-containing protein.

**Table 3:** List of candidate genes

**QTL**	**Candidate genes**	***Prunus Persica* homolog**	***Arabidopsis thaliana* homolog**	**Uniprot function prediction**
**qP-CRO2**	*Prudul26A013473*	*Prupe.2G196600*	*AT3G61910 AT2G46770*	NAC domain-containing protein
**qP-DK7.1**	*Prudul26A012082*	*Prupe.7G052700*	*AT3G57290*	Eukaryotic translation initiation factor 3 subunit E
	*Prudul26A002885*	*Prupe.7G052800*	*AT4G21000 AT4G20990*	Alpha-carbonic anhydrase domain-containing protein
**qP-DK7.2**	*Prudul26A005959*	*Prupe.7G092000*	*AT5G06470*	Uncharacterized protein
	*Prudul26A029836*	*Prupe.7G092100*	*AT5G06480 AT3G11780*	ML domain-containing protein
	*Prudul26A017782*	*Prupe.7G092200*	*AT2G18400*	Ribosomal_L6 domain-containing protein
	*Prudul26A008330*	*Prupe.7G092300*	*-*	Uncharacterized protein
**qP-BLO2**	*Prudul26A000954*	*Prupe.2G169700*	*AT4G00520 AT1G01710*	Cyclic nucleotide-binding domain-containing protein
	*Prudul26A028547*	*Prupe.2G169800*	*AT3G49050*	Uncharacterized protein
	*Prudul26A019171*	*Prupe.2G169900*	*AT2G45880*	Beta-amylase

The length of the qP-DK7.1 region was 11.5 kb, containing two genes (Table 3) encoding eukaryotic translation initiation factor 3 subunit E and an alpha-carbonic anhydrase domain-containing protein.

The length of qP-DK7.2 was 11.5 kb, containing four genes (Table 3) encoding an ML domain-containing protein, a ribosomal L6 domain-containing protein, and two uncharacterized proteins.

The length of qP-BLO2 was 13.8 kb, containing three genes (Table 3) encoding a cyclic nucleotide-binding domain-containing protein, a beta-amylase, and an uncharacterized protein.

**Figure 4:**
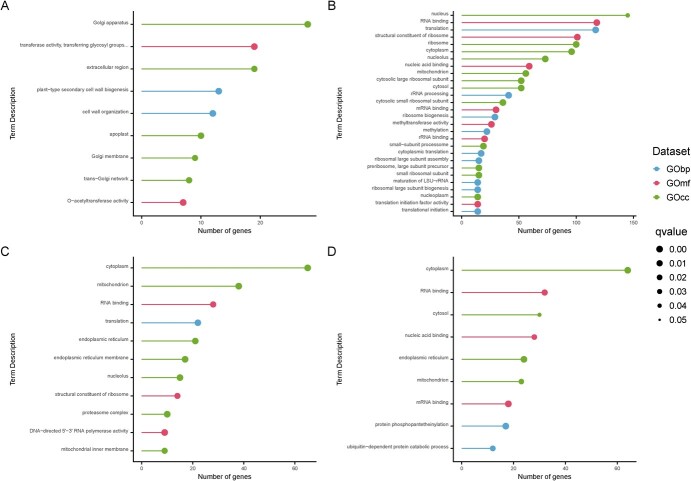
Top enriched terms of the most suitable candidate genes. A) *Prupe.2G196600* B) *Prupe.2G169700* C) *Prupe.7G052700* D) *Prupe*.*7G092200*.

The gene coexpression network (GCN) analysis of *Prupe.2G196600*, the homologous gene of *Prudul26A013473*, indicated 25 enriched terms, with nine classified as top enriched terms (TET) (Supplementary Material 2; Figure 4A). Within GObp, we found two TETs: ‘*plant-type secondary cell wall biogenesis’* and *‘cell wall organization’*. Within GOmf, we found two TETs: ‘*transferase activity, transferring glycosyl groups’* and *‘O-acetyltransferase activity’*. Within GOcc, we found five TETs: ‘*Golgi apparatus’*, ‘*extracellular region’, ‘apoplast’,* ‘*Golgi membrane’,* and *‘trans-Golgi network’*.

The GCN analysis of *Prupe.7G052700*, the homologous gene of *Prudul26A012082*, indicated 192 enriched terms, and 28 were classified as TETs (Supplementary Material 2; Figure 4B). Within GObp, we found nine TETs: ‘*translation’, ‘rRNA processing’, ‘ribosome biogenesis’, ‘methylation’, ‘cytoplasmic translation’, ‘ribosomal large subunit assembly’, ‘maturation of LSU-rRNA’, ‘ribosomal large subunit biogenesis’,* and *‘translational initiation’*. Within GOmf, we found seven TETs: ‘*RNA binding’, ‘structural constituent of ribosome’, ‘nucleic acid binding’, ‘mRNA binding’, ‘methyltransferase activity’, ‘rRNA binding’,* and *‘translation initiation factor activity’*. Within GOcc, we found 12 TETs: ‘*nucleus’, ‘ribosome’, ‘cytoplasm’, ‘nucleolus’, ‘mitochondrion’, ‘cytosolic large ribosomal subunit’, ‘cytosol’*, ‘*cytosolic small ribosomal subunit’, ‘*small*-subunit processome’, ‘preribosome large subunit precursor’, ‘small ribosomal subunit’,* and *‘nucleoplasm’*.

The GCN analysis of *Prupe.7G092200*, the homologous gene of *Prudul26A017782*, indicated 161 enriched terms, and 11 were classified as TETs (Supplementary Material 2; Figure 4C). Within GObp, we found one TET: ‘*translation’.* Within GOmf, we found three TETs: ‘*RNA binding’, ‘structural constituent of ribosome’* and *‘DNA-directed 5′′-3′ RNA polymerase activity’*. Within GOcc, we found seven TETs: ‘*cytoplasm’, ‘mitochondrion’, ‘endoplasmic reticulum’, ‘endoplasmic reticulum membrane’, ‘nucleolus’, ‘proteasome complex’,* and *‘mitochondrial inner membrane’*.

The GCN analysis of *Prupe.2G169700*, the homologous gene of *Prudul26A000954*, indicated 32 enriched terms, with nine classified as TETs (Supplementary Material 2; Figure 4D). Within GObp, we found two TETs: ‘*protein phosphopantetheinylation’* and *’ubiquitin-dependent protein catabolic process’*. Within GOmf, we found three TETs: ‘*RNA binding’, ‘nucleic acid binding’,* and *‘mRNA binding’*. Within GOcc, we found four TETs: ‘*cytoplasm’, ‘cytosol’, ‘endoplasmic reticulum’,* and *‘mitochondrion’*.

## Discussion

### Almond genetic structure explains its historical worldwide dissemination

Our results strongly supported the subdivision of these accessions into five ancestral groups. Each group was formed by accessions with a common geographical origin: G2 exclusively by Spanish accessions, while G1, G3, G4, and G5 were mainly formed by Chinese, Italian, American and accessions, respectively. These results are in agreement with Pavan et al. 2021, who, with a more limited germplasm, found four ancestral groups, each formed mainly by accessions from Spain, France, Italy and the USA.

Apart from Chinese accessions, G1 included accessions from Pakistan, Iran, Turkey, Greece, and Romania. Due to the diverse origin of the accessions forming this group, they may be considered as part of a more primitive almond pool. This agrees with the first dissemination of almond from its center of origin, spreading throughout south-western Asia, eventually reaching modern Turkey, Greece, and other regions of the Eastern Mediterranean [26].

After reaching the Eastern Mediterranean, Greeks, and Phoenicians introduced the almond to other adapted areas of the Mediterranean. By the time of the Roman Empire, almond cultivation had spread all along the Mediterranean coast. Ancestral groups G2, G3, and G5 may have been established during this period.

G2 was entirely formed by Spanish accessions. The fact that the Spanish accessions were related to Tunisian and other North African accessions could be explained by the introduction of new North African genetic material during the Arab occupation of the Iberian Peninsula.

Even though almond cultivation was introduced in California by the Spanish missions, our results showed a close genetic relationship between Californian and French accessions. This was noticeable in both phylogenetic tree, kinship, and population structure analysis. In the phylogenetic tree, all Californian accessions were located in only one branch, clustering together with the French accessions. In the kinship analysis, both G4 and G5 clusters were connected by several edges, being the clusters with most connections between each other, indicating a relatively recent common ancestor between these two groups. Finally, the population structure analysis included two French accessions, ‘Princesse789’ and ‘A la Dame (CG14)’ within group G4. This genetic relatedness between French and Californian accessions is explained by the introduction of French commercial cultivars in California from 1850 to 1900 [27]. In this sense, ‘Princesse789’ and ‘A la Dame (CG14)’ may be among the cultivars introduced in California during that period, or at least, close relatives.

G5 formation also included three Tunisian accessions. This close genetic relatedness may be explained by the exchange of material between the two countries during the French occupation of Tunisia from 1881 to 1956.

Finally, Californian cultivars were introduced to adapted regions of the southern hemisphere, including Chile, Argentina, South Africa, and Australia. This explains the genetic relatedness between Californian and Australian accessions.

### The cultivated almond shows signs of inbreeding and domestication

Homozygosity analysis was used to study the inbreeding levels in the studied germplasm. Different *ROH* lengths allowed us to detect modern (*ROH_2_*) and ancient (*ROH_0.25_*) inbreeding events. In general, all accessions had a low *F_0.25_* and *F_2_* value, regardless of whether they were classified as landraces or breeding cultivars. Nevertheless, there were some breeding cultivars with an *F_2_* value over 0.4, such as ‘Ayles’, ‘Amandier rose’, and ‘Garfi’. These results are in agreement with [28], who concluded that breeding practices could be increasing inbreeding levels.

Looking at *Freq_0.25_* and *Freq_2_*, we found no region with high *Freq_2_*. On the other hand, there were several regions with a high *Freq_0.25_*, in chromosomes 1, 4, and 5. This may respond to selection pressure during the almond domestication process, fixing in the genome alleles of interest. In the case of chromosome 4, the peak of high *Freq_0.25_* was formed only by one gene, *Prudul26A014015*. According to phylomeDB, *Prudul26A014015* had two homologous genes in Arabidopsis, *AT1G21390* and *AT1G76980*. *AT1G21390* was annotated as *EMBRYO DEFECTIVE* (*EMB*) *2170*, while *AT1G76980* was annotated as patatin-like phospholipase domain protein. *EMB* genes are related with embryo development in Arabidopsis [29]. Since the edible commercial product of the almond is the seed, selective pressure may be exerted on a gene related with embryo and seed development.

### Non-additive GWAS, short LD decay and GCN analysis allowed the discovery of several candidate genes for breeding traits

Among the 13 QTLs detected in this study, only one had an additive effect. This indicates that non-additive effects could be the main source of genotype–phenotype interactions in almond. Furthermore, due to the similarity between *Prunus* genus genomes, this phenomenon could be repeated in other cross-pollinating *Prunus* species. In peach, a self-compatible *Prunus* species that still maintains an important level of heterozygosity, this may have predominantly led to the fixation of dominant/recessive QTLs and to the selection for heterozygosis in those that are overdominant.

Another remarkable aspect was that most of the QTLs detected in this study were defined by only one SNP (the top SNP). This is caused by the short LD decay found in the population, affecting this study in two different ways: first, it has facilitated the search for candidate genes, since the genomic regions associated with the traits of interest were small and only a few genes were found in these regions (in the case of qP-CRO2, only one gene was found in the QTL region). Second, this could have limited the detection of some regions of interest. In genomic regions with a lower concentration of SNPs, some regions of interest might have been lost because the distance between SNPs was greater than the LD decay. This could be the case for KW, were no QTL was found.

For the rest of the traits under study, we found several QTLs that could be used in marker assisted selection. However, the mathematical models used to calculate the combined variance explained by these QTLs did not take into account possible epistatic interactions between the QTLs. More research is needed before these QTLs can be used in marker assisted selection.

Among the 13 QTLs detected in this study, only qP-CRO2 has been reported in other studies [20, 25, 30, 31]. Only one gene was found in the qP-CRO2 region, *Prudul26A013473*. This gene, annotated as a NAC TF, was homologous to *NST1* in Arabidopsis. *NST1* has been reported as a key regulator of the formation of secondary cell walls in woody tissues [32] and specifically associated with secondary cell wall formation within the enb layer in Arabidopsis seeds [33]. Furthermore, the GCN analysis of *Prupe.2G196600*, the homologous gene of *Prudul26A013473*, gave several enriched terms related to secondary cell wall biogenesis and organization. All this evidence indicates that *Prudul26A013473* is the gene responsible for qP-CRO2, having a major role in the transcriptional regulation of the almond endocarp lignification.

For double kernels percentage, we found two different QTLs, qP-DK7.1, and qP-DK7.2. In almond, the presence of double kernels in a shell is due to the development and fertilization of two ovules in the ovary when the secondary ovule does not degenerate [34]. In the case of qP-DK7.1, only two candidate genes were found in the QTL region, *Prudul26A012082* and *Prudul26A002885*. These genes were annotated as a eukaryotic translation initiation factor 3 subunit E (*eIF3e*) and alpha-carbonic anhydrase domain-containing protein, respectively. *eIF3e* has been reported as essential for embryo development and normal plant cell growth in Arabidopsis [35, 36]. The GCN analysis of *Prupe.7G052700*, the homologous gene of *Prudul26A012082*, gave several terms related to embryo development (Supplementary Material 2). This indicates that *Prudul26A012082* is the gene responsible for qP-DK7.1, having a major role in almond ovule and embryo development.

There were four candidate genes in qP-DK7.2 region, *Prudul26A029836*, *Prudul26A017782, Prudul26A005959,* and *Prudul26A008330*. These genes were annotated as a ML domain-containing protein, a ribosomal large subunit 6 (*RL6*) and two uncharacterized proteins, respectively. It has been suggested that *RL6*, among other ribosomal subunits, is essential for embryogenesis in Arabidopsis [37]. The GCN analysis of *Prupe.7G052700*, homologous gene of *Prudul26A012082*, showed several terms related to cell cycle regulation and cell development (Supplementary Material 2): *Prudul26A017782* is most likely the gene responsible for qP-DK7.2, having a major role in almond ovule and embryo development.

Within qP-BLO2, three genes were found, *Prudul26A000954*, *Prudul26A028547,* and *Prudul26A019171*. These genes were annotated as a cyclic nucleotide-binding domain-containing protein, an uncharacterized protein and a beta-amylase. Both *AT1G01710* and *AT4G00520*, the homologous genes of *Prudul26A000954* in Arabidopsis, were annotated as Acyl-CoA thioesterases. These proteins catalyze the hydrolysis of acyl-CoAs to free fatty acids and coenzyme A. During dormancy breaking in perennial fruit trees, reactive oxygen species (ROS) are produced. One of the pathways that produces these ROS starts from fatty acids, beta-oxidated to monosaccharides, and these monosaccharides produce ROS via mitochondrial respiration or are oxidized via the Pentose Phosphate Pathway [38]. The GCN analysis of *Prupe.2G169700*, the homologous gene of *Prudul26A000954*, gave several terms related with SWI/SNF complex and BAF60 (Supplementary Material 2). SWI/SNF complexes have been shown to participate in the control of flower development and blooming time [39]. BAF60 is a SWI/SNF subunit, and induces a change at the high-order chromatin level, repressing the photoperiod flowering pathway in Arabidopsis [40]. *Prudul26A000954* therefore appears to be the gene responsible for qP-BLO2, having a major role in blooming time through fatty acids metabolism.

## Conclusions

In this study, we carried out genetic structure analysis and non-additive GWAS in a set of 243 almond accessions. Our genetic results agreed with the archaeological and historical evidence that separate modern almond dissemination into four phases: Asiatic, Mediterranean, Californian, and southern hemisphere. Of the 13 QTLs found for the traits of interest, only one had an additive effect, suggesting that non-additive effects could be the major source of genotype–phenotype interactions in almond and other *Prunus* species. Based on the fast LD decay and the use of the peach GCN we propose four candidate genes for the main QTLs found in this study.

## Materials and methods

### Plant material and genotyping

We used a diversity panel of 243 accessions from 21 countries and five continents (Supplementary Material 3). Of the 243 accessions, 161 were maintained in the INRAE collection (43.948611 N, 4.808333 E) and 97 in the IRTA collection (41.170723 N, 1.172942 E), with 78 accessions in common at the two locations. DNA of the 180 accessions from the INRAE and IRTA collections was extracted from leaves according to [41]. After DNA extraction, samples were genotyped using the 60 K SNP array [24].

For the remaining 63 accessions, genotype information was obtained from two different sources: 45 resequences were from a previous study [24] and resequences from 18 accessions were downloaded from NCBI (Supplementary Material 3).

### Genotypic data filtering and datasets

SNP calling of samples from the DNA libraries was according to Duval et al. 2023. Only SNPs present in the 60 K almond SNP array were selected (60 581 SNPs). All the samples were merged, giving a dataset with 243 accessions and 60 581 SNPs. These SNPs were filtered following these criteria: i) call rate per sample higher than 82%, ii) call rate per SNP higher than 90%, and iii) minimum allele frequency (MAF) higher than 5%.

Using this initial dataset (Table 4), we calculated the identity-by-state, i.e. the number of SNPs with the same allelic state shared between accessions. Accessions with an identity-by-state higher than 98% were declared clonal groups. In total, 22 clonal groups with two or more accessions were detected. Within each clonal group, the accession with the highest number of SNPs was selected (Supplementary Material 3). The remaining accessions were classified as landraces and breeding cultivars based on pedigree information (Supplementary Material 3).

**Table 4:** Description of the four datasets used

Dataset	**N° accessions**	**Accessions included**	**N° SNPs**
Initial	243	All	54 112
Structure	152	Classified as landraces	53 985
Nut traits	79	Phenotyped for nut traits	22 928
Blooming time	167	Phenotyped for blooming time	16 804

From the initial dataset, three more datasets were created for each analysis in this study. For the genetic structure analysis, only the 152 accessions classified as landraces were selected. We also created two more data sets for GWAS, including only phenotyped individuals for nut traits and blooming time, with 79 and 167 accessions respectively. After selecting the accessions, the datasets were filtered again following the same criteria described above. For the datasets used in GWAS, we included two more criteria: (iv) SNPs with three genotypic classes, (v) minimum genotypic class frequency higher than 5%. The four datasets used in this study are presented in Table 4.

### Genetic structure analysis

A population structure analysis, an additive kinship, a phylogenetic tree and a principal component analysis (PCA) were used to determine the genetic structure of the 152 accessions classified as landraces. The population structure analysis was performed using the LEA R package [42]. The number of ancestral groups tested were from 1 to 15 with ten repetitions. An accession was considered to belong to an ancestral group when the coefficient of belonging to that specific group was higher than 60%. If an accession did not belong to any ancestral group, it was considered admixed. Additive kinship was estimated with the rrBLUP R package [43]. The phylogenetic tree was built using the unweighted pair group method with arithmetic mean algorithm included in the ape R package [44], and PCA using the factoMineR R package [45].

### Homozygosity analysis

Runs of homozygosity (*ROHs*) were analyzed in all 243 accessions using the detectRUNS R package (https://cran.r-project.org/package=detectRUNS). Two lengths of *ROHs* were analyzed: *ROH_2_* higher than 4 163 686 bp and *ROH_0.25_* higher than 520 461 bp (2% and 0.25% of the almond genome size according to ‘Texas’ reference genome v2.0 [22], respectively). *ROH_2_* and *ROH_0.25_* were detected using a window size equal to 20 SNPs. The maximum gap between SNPs was equal to 1 000 000 bp for *ROH_2_* and 100 000 bp for *ROH_0.25_*. For every accession, the overall inbreeding values *F_2_* and *F_0.25_* were calculated using *ROH_2_* and *ROH_0.25_*, respectively. Finally, we calculated the frequencies *Freq_2_* and *Freq_0.25_* with which every SNP was located in a *ROH_2_* and *ROH_0.25_*, respectively.

### Linkage disequilibrium decay

The squared correlated coefficient, r^2^, was estimated in the 152 individuals classified as landraces using VCFTools v0.1.16 [46]. As it was calculated individually for every chromosome using a 250 000 bp window, the r^2^ was calculated for every combination of SNPs within that window. We used a threshold of 0.2 to set the LD decay which was then represented graphically using a loess regression function with a span of 0.1.

### Phenotypic data collection and analysis

As nut traits, we phenotyped nut weight (NW), kernel weight (KW), crack-out percentage (CRO) and double kernels percentage (DK). Each accession was phenotyped between 9 and 12 years in the IRTA collection. From each accession, at least 100 mature fruits were randomly collected. The fruit was considered mature when the mesocarp was fully dry and split along the fruit suture and the peduncle was near to complete abscission. Samples were stored at room temperature for at least 2 weeks. After measuring NW, the shells were cracked to measure the weight of the kernels. All weights were measured using an electronic balance. DK was measuring by counting the number of shells containing double kernels. CRO was calculated according to Equation 1:

(1) \begin{equation*} CRO=\left( NW- KW\right)/ NW \end{equation*}

Blooming time (BLO) was phenotyped for three consecutive years (2020–22) as Julian days when about 5% of flower buds were fully open for each tree. This trait was measured in the INRAE collection.

Best Linear Unbiased Prediction (BLUP) for NW, KW, CRO, and BLO was estimated for each genotype using a linear mixed model according to Equation 2:

(2) \begin{equation*} {P}_{ijk}=\mu +{Y}_{ik}+{G}_j+{e}_{ijk} \end{equation*}

Where ${P}_{ijk}$ is the phenotypic value (=BLUP) of the kth repetition of the jth genotype in the ith year, μ is the mean value of the phenotypic trait, ${Y}_{ik}$ is the fixed effect of the kth repetition of the ith year, ${G}_j$ is the random genotypic effect of genotype j, and ${e}_{ijk}$ is the residual error of the model.

BLUP for DK was estimated for each genotype using a linear mixed model according to Equation 3:

(3) \begin{equation*} {P}_{jk}=\mu +{G}_j+{e}_{jk} \end{equation*}

where for equation 2, ${P}_{jk}$ is the phenotypic value of the kth repetition of the jth genotype, μ and ${G}_j$ have the same meanings as in equation 1, ${e}_{jk}$ is the residual error.

For every trait, broad-sense heritability (${h}^2$) was estimated as:

$$ {h}^2=\frac{{\sigma^2}_G}{{\sigma^2}_G+\frac{{\sigma^2}_{\varepsilon }}{n}} $$

where ${\sigma^2}_G$ is the genotype variance, ${\sigma^2}_{\varepsilon }$ is the residual variance and $n$ is the mean number of measures.

### Genome-wide association study (GWAS)

We explored additive and non-additive genotype–phenotype associations in two different datasets: nut traits and blooming time datasets, with 79 and 167 accessions respectively. For this purpose, we transformed these genotypic datasets as follow. For additive effects, the three possible genotypes of a biallelic marker with a reference allele (*a1*) and an alternative allele (*a2*), were written in numeric representation as 1 (*a1a1*, homozygous for the reference allele), 0 (*a1a2*, heterozygous) and −1 (*a2a2*, homozygous for the alternative allele). For dominant effects, genotypes *a1a1* and *a1a2* have the same effect in the phenotype, so *a1a1* and *a1a2* were codified as 1 and *a2a2* as −1. For recessive effects, genotypes *a1a2* and *a2a2* have the same effect in the phenotype, so *a1a2* and *a2a2* were codified as −1 and *a1a1* as 1. Note that the dominant and recessive transformations correspond to a dominant-recessive genotype–phenotype interaction, but we had to differentiate the effects of a dominant reference allele or a dominant alternative allele. For overdominant effects, genotypes *a1a1* and *a2a2* have the same effect in the phenotype, so genotypes *a1a1* and *a2a2* were codified as 1 and *a1a2* as 0 (Supplementary Material 1) [7].

The mixed model from rrBLUP R package [43] was used in this study. BLUPs were used as phenotypic data for each trait. For every model, we used three different corrections: including the additive kinship (K), the population structure (Q) or both (K + Q):

(4) \begin{equation*} Y=\mu+X\beta+Qv+Zu+\varepsilon \end{equation*}

where Y is the vector of phenotypic values, μ the overall mean, X the allelic state matrix, β the allelic effect of each SNP 4, Q is the structural matrix estimated by the LEA R package, v is an effect vector estimated by the model and used as a fixed effect, Z is an incidence matrix linking observations to the vector u that is a polygenic random effect with a covariance structure defined by the kinship (K as previously estimated) u ~ N (0;2KVg), and ε the residual effect.

The choice of each correction was based on the adjustment of the p-values obtained to a uniform distribution as expected under the null hypothesis. The corrected Bonferroni threshold at 5% was used to identify significant association between phenotypic data and genotypic markers.

Before considering any significant genotype–phenotype association found as a QTL, we used visual analysis to confirm that the phenotypic data distribution matched the genotype–phenotype interaction searched (e.g., if an association found with the additive transformation matched an additive phenotypic distribution). We considered any significant genotype–phenotype association matching its phenotypic data distribution as a true positive QTL.

For the QTLs considered as true positives, we assumed the Simple model’s R^2^ as the variance explained for those QTL. We also calculated the combined variance of the QTLs detected for every trait. In this case, we assumed as the combined variance explained the R^2^ of a linear regression using all the top SNPs detected for a trait.

### Candidate gene analysis

For every trait, we selected the QTL with the highest -log10(p-value). If the QTL selected had a -log10(p-value) higher than 6.5, we performed a candidate gene analysis. In the case of DK, we used candidate gene analysis on the two QTLs we found, as both had a -log10(p-value) higher than 6.5.

Every QTL region was defined using the position of the top SNP and the estimated LD decay for every chromosome. The beginning of the QTL was defined as the top SNP position minus the estimated LD decay and the end of the QTL was defined as the top SNP position plus the estimated LD decay. We determined the number of genes located in the QTL region using the ‘Texas’ reference genome v2.0 [22]. Any gene located less than 2000 bp from the QTL region was included in it, as we considered that the regulatory region of that gene was situated within the QTL region. Then we searched the homologous genes from peach (*Prunus persica*) and Arabidopsis (*Arabidopsis thaliana*).

To obtain more information on the function of the most suitable candidate genes, *Prudul26A013473*, *Prudul26A012082*, *Prudul26A017782*, and *Prudul26A000954*, we used the PeachGCN v1 [47] for gene coexpression network analysis. We first determined the homologous of our candidate genes in peach, then extracted coexpressing genes. Finally, we performed an enrichment analysis of the coexpressing subnetworks using Gene Ontology (GO) and Mapman ontologies [48, 49]. The significance threshold was held at q-value <0.05. Enriched terms annotating at least 2% of the genes in the coexpressing subnetworks were classified as top enriched terms (TET).

## Supplementary Material

Web_Material_uhad193

## Data Availability

All resequences used in this study can be found at NCBI under the following accession numbers: SRR3141040, SRR3141049, SRR3141057, SRR3141065, SRR3141073, SRR3141083, SRR3141098, SRR3141113, SRR3141181, SRR3141192, SRR3141204, SRR3141229, SRR4036105, SRR4045224, SRR4045225, SRR4045227, SRR4045228, SRR4045229.
