# Supplementary material for: Almond population genomics and non-additive GWAS reveal new insights into almond dissemination history and candidate genes for nut traits and blooming time
Source: Hortic Res. 2023 Sep 25;10(10):uhad193. doi: 10.1093/hr/uhad193 (PMC10623407; doi:10.1093/hr/uhad193)
Supplement: Web_Material_uhad193 [file web_material_uhad193.zip › Supplementary Material 1.docx]

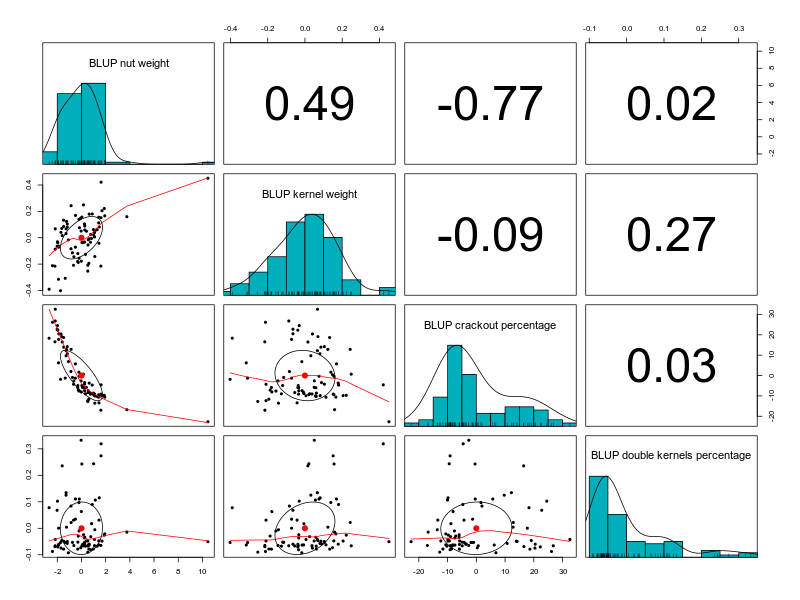


**Supplementary Figure 2***.* Correlation between traits.


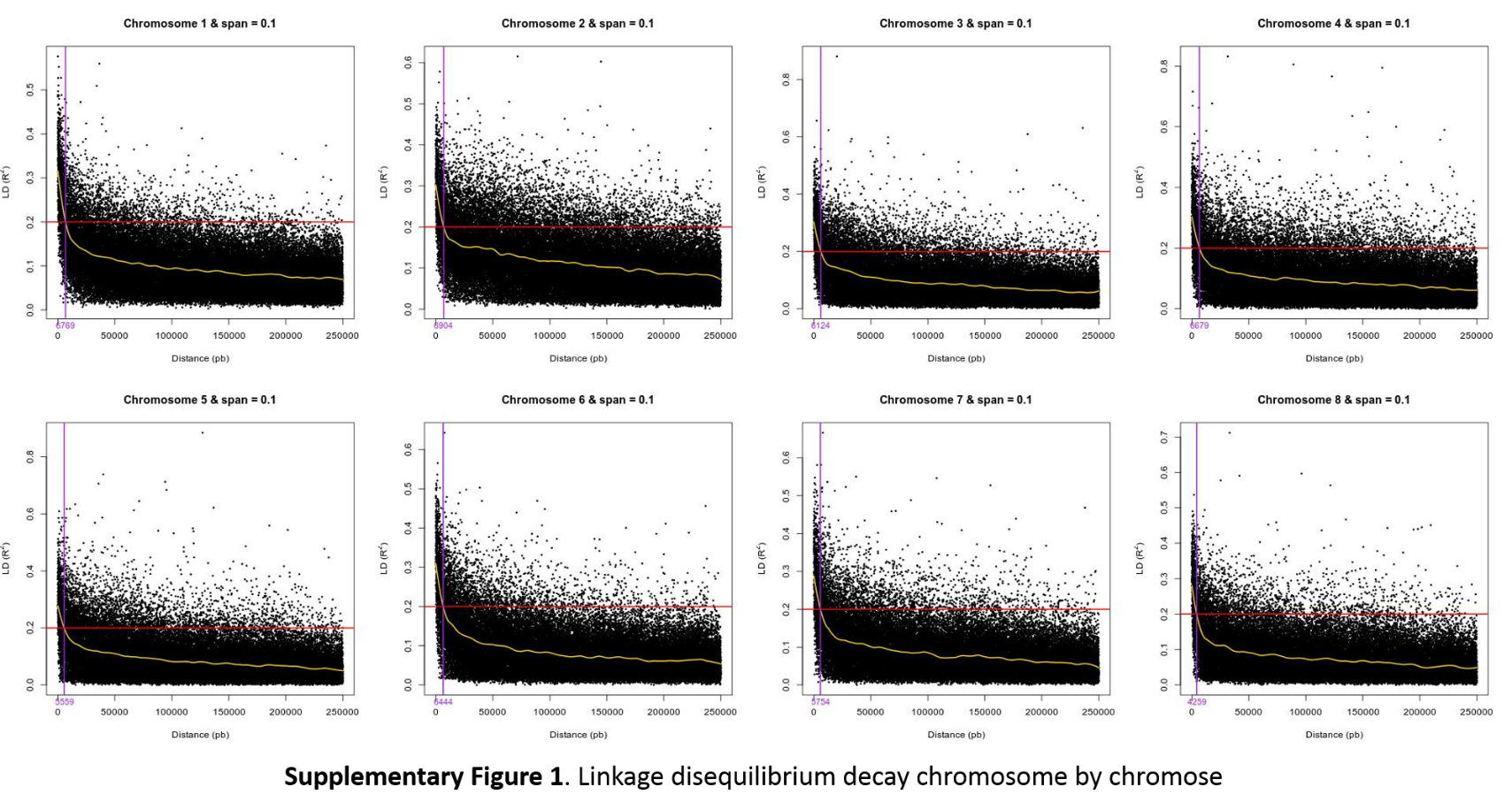


**Supplementary Figure 1***.* Linkage disequilibrium decay chromosome by chromosome.


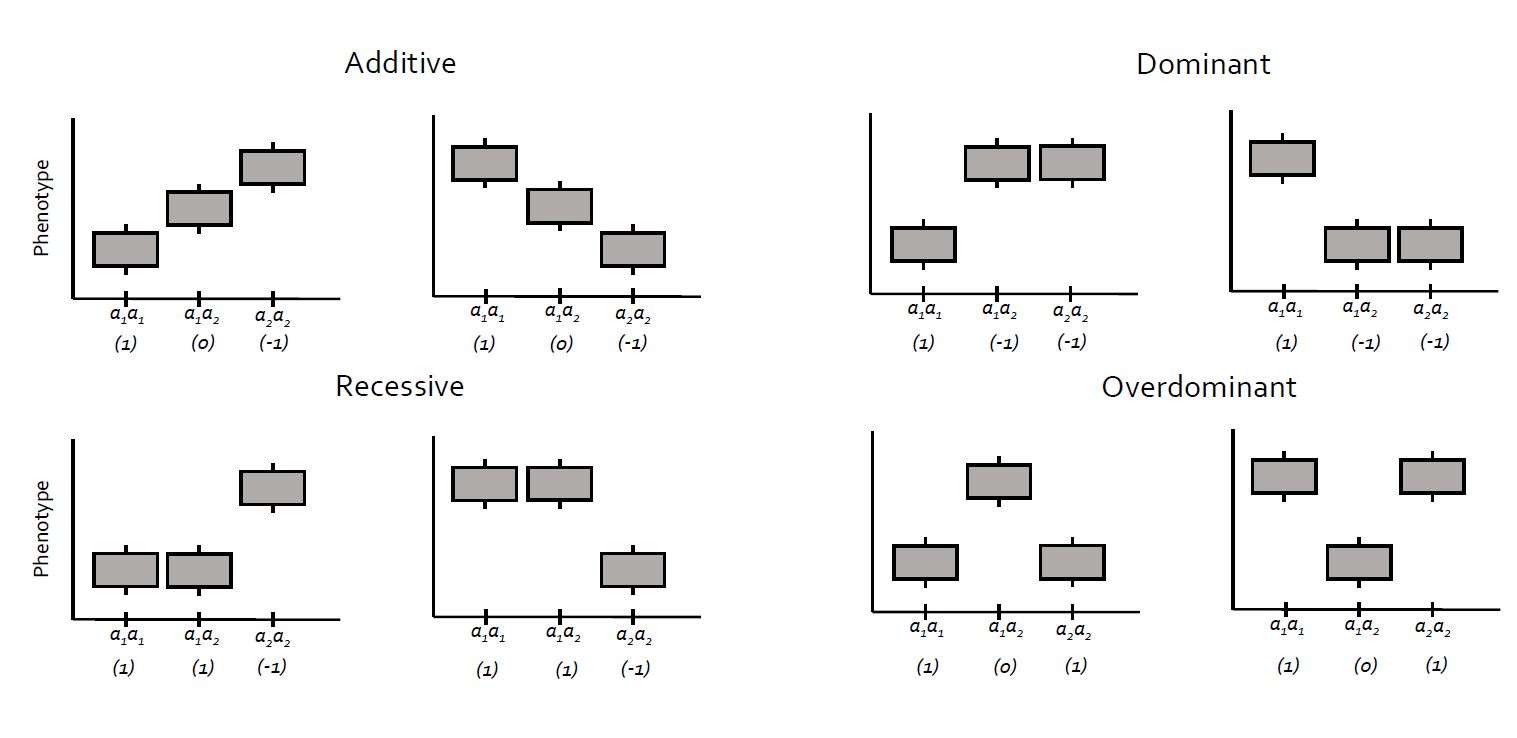


**Supplementary Figure 3***.* Additive and non-additive genotype-phenotype associations.
